# Supplementary material for: The Genetic Complexity of Type-IV Trichome Development Reveals the Steps towards an Insect-Resistant Tomato
Source: Plants (Basel). 2022 May 14;11(10):1309. doi: 10.3390/plants11101309 (PMC9148003; doi:10.3390/plants11101309)
Supplement: Supplementary file 1 [file plants-11-01309-s001.zip › plants-11-01309-s001/Vendemiatti et al_Plants_Suppl_Apr_12_22.pdf]

Table S1. Oligonucleotide sequences used in this work.

Primer	Sequence	Gene Model	Utilization	Reference
1B	AGCACTTATGGGCCTGTTCA<br>CAGCGCATCTTGAGTGTTGT	Solyc01g088100	CAPS marker	This work
2A	ACAACGACTAGAACGGCTGG<br>CCCATCACAACCTAATGTCTAAGG	Solyc02g092570	CAPS marker	This work
2B	GCACAACATCCACCCTTCTT<br>CAAGCACATACGATTGCAGA	Solyc02g093030	CAPS marker	This work
2C	GAAGGGTCGGGTACAAGGTAA<br>CCACTCTCTTAGCCTGAATGG	Solyc02g094500	CAPS marker	This work
3A	GCTCTGAAGATCCTACACATGAAA<br>TCCCATGCTCTTTCTGGAAC	Solyc03g019800	CAPS marker	This work
3B	CGGCGTAAGACAAACACGTTC<br>GCATTATCAACTCATCTACCAGTCC	Solyc03g083830	CAPS marker	This work
3C	TTGGGAGATTAGCGTAAGGAG<br>ATAGGAGCAACAAGGTACATGC	Solyc03g093610	CAPS marker	This work
6A	GCAGTGTGATGCTGGTGTTT<br>AGGCGACTTGGTTGTCACTC	Solyc06g005060	CAPS marker	This work
6B	TGTTGTTTCCCTTGTTGCAG<br>GCCAGTTTGAACCAGACCAG	Solyc06g009730	CAPS marker	This work
6C	AGCTAAGATGGAGGGAGATGC<br>CCCGAGGTGTAAACTGTAACG	Solyc06g048460	CAPS marker	This work
6D	AAAACACGGCGATGAGAAAT<br>TTTCCTCTCCAATTCCCAAT	Solyc06g060210	CAPS marker	This work
FW2.2	GGTGGTGTTGATGTGGAGTGAGTG<br>GGCAGATACATAGTGAGGAGGAAC	Solyc02g090730	CAPS marker	This work
DWARF	TGATCCATATTCGTTCAATCCA<br>CGTGATTATGTTAGCGGGAAT	Solyc02g089730	CAPS marker	This work

FW3.2	GAGATAACGGGTTAAATAGAGT<br>TAGTTAGGATAGTTATAGTTTGC	Solyc03g114940	CAPS marker	This work
EJ-2	CACAATTCATGCTGGATCAGC<br>CGGAGTAATCTATTAGATTCTGC	Solyc03g114840	CAPS marker	This work
SP5G	TAATTACGCAGTGACGAAGCA<br>TTGACACAGAGTTCGAGAACG	Solyc05g053850	CAPS marker	This work
SP	GGGTTGAAGTTCATGGTGGT<br>AGTGCCTGGAATGTCTGTGA	Solyc06g074350	CAPS marker	This work
PTS	AGGAAGTGATTGACCCATGC<br>CCCCAAACACCACTATCTAAGC	Solyc06g072480	CAPS marker	This work
ASAT1	GGGAGGCCAAGACAAGTTGATA<br>TGGAGAAGCAAACTGAAGAAAATC	Solyc12g006330	qPCR	[12]
ASAT2	GACTCCATTCGTCCATCTTTACTTC<br>TTTGACTTCTTCCTTCTCCTTTCTTA	Solyc04g012020	qPCR	
ASAT3	TTTCTTCCCTTTACCGTCTGAA<br>TGAACAAGTGCTGAGGCAAC	Solyc11g067270	qPCR	This work
ASAT4	GGTGGTCGTGATGTCCCTAA<br>GCCCTCCTTGTTAGCAGTTG	Solyc01g105580	qPCR	This work
ASH1	TCTTTCATCCAACGTGATTAACATTT<br>ATCTACACCACAGAACACTACCAATA	Solyc05g051660	qPCR	This work
ASH2	GCGACCCACTGAATAGCATC<br>GGCGGAGGATTTGTATTAGGA	Solyc05g051670	qPCR	This work
ASH3	CTACACTCAAATCAACTTCCATACCATACA<br>ATAGAACCGTTCGACTCGACCATTT	Solyc09g075710	qPCR	[51]
ABC transporter	TCCGAAGGGATGATGGAG<br>GCAGAAGACCAAATACAGGGTAA	Solyc03g005860	qPCR	This work
ACTIN	GGTCCCTCTATTGTCCACAG<br>TGCATCTCTGGTCCAGTAGGA	Solyc04g011500	qPCR	[71]

EF1α	AAGCCCATGGTTGTTGAGAC<br>TTCTTGACAACACCCACAGC	Solyc06g005060	qPCR	[72]

References

1. Fan, P.; Miller, A.M.; Schilmiller, A.L.; Liu, X.; Ofner, I.; Jones, A.D.; Zamir, D.; Last, R.L. In Vitro Reconstruction and Analysis of Evolutionary Variation of the Tomato Acylsucrose Metabolic Network. *Proc. Natl. Acad. Sci. U.S.A.* **2016**, *113*, doi:10.1073/pnas.1517930113.
2. Schilmiller, A.L.; Gilgallon, K.; Ghosh, B.; Jones, A.D.; Last, R.L. Acylsugar Acylhydrolases: Carboxylesterase-Catalyzed Hydrolysis of Acylsugars in Tomato Trichomes. *Plant Physiol.* **2016**, *170*, 1331–1344, doi:10.1104/pp.15.01348.
3. Silva, N.C.Q.; de Souza, G.A.; Pimenta, T.M.; Brito, F.A.L.; Picoli, E.A.T.; Zsögön, A.; Ribeiro, D.M. Salt Stress Inhibits Germination of Stylosanthes Humilis Seeds through Abscisic Acid Accumulation and Associated Changes in Ethylene Production. *Plant Physiology and Biochemistry* **2018**, *130*, 399–407, doi:10.1016/j.plaphy.2018.07.025.
4. Pinto, M. de S.; Abeyratne, C.R.; Benedito, V.A.; Peres, L.E.P. Genetic and Physiological Characterization of Three Natural Allelic Variations Affecting the Organogenic Capacity in Tomato (Solanum lycopersicum cv. Micro-Tom). *Plant Cell Tiss Organ Cult* **2017**, *129*, 89–103, doi:10.1007/s11240-016-1159-2.

Table S2. Genomic coordinates (ITAG2.5) of the genetic variation from *S. galapagense* present at high frequencies (≥0.8) in the *Get*-like phenotypical group of the MT-*Get* segregating population.

Chromosome	Corresponding Region (bp)
**1**	82,558,349 – 83,597,817
**2**	53,604,856 - 55,297,272
**3**	61,495,951 - 65,007,167
**5**	19,540,000 - 19,637,400
**6**	3,688 - 38,647,717

Table S3. GC-MS content of acyl groups in *S. galapagense*, MT-*Get* and Micro-Tom (MT).

RT	Acyl Groups Content	Genotypes		
		S. galapagense	**MT-*Get***	**MT**
1	2-Methyl Butanoate	0.77 ± 0.30	0.00	0.00
2	3-Methyl Butanoate	2.86 ± 1.20	0.00	0.00
3	Methyl Decanoate	0.18 ± 0.07	0.00	0.00
4	Methyl Dodecanoate	2.92 ± 0.90	0.06 ± 0.00	0.00

Data are means (n =8) ± SD.
