# Supplementary figures and images for: The Genetic Complexity of Type-IV Trichome Development Reveals the Steps towards an Insect-Resistant Tomato
Source: Plants (Basel). 2022 May 14;11(10):1309. doi: 10.3390/plants11101309 (PMC9148003; doi:10.3390/plants11101309)

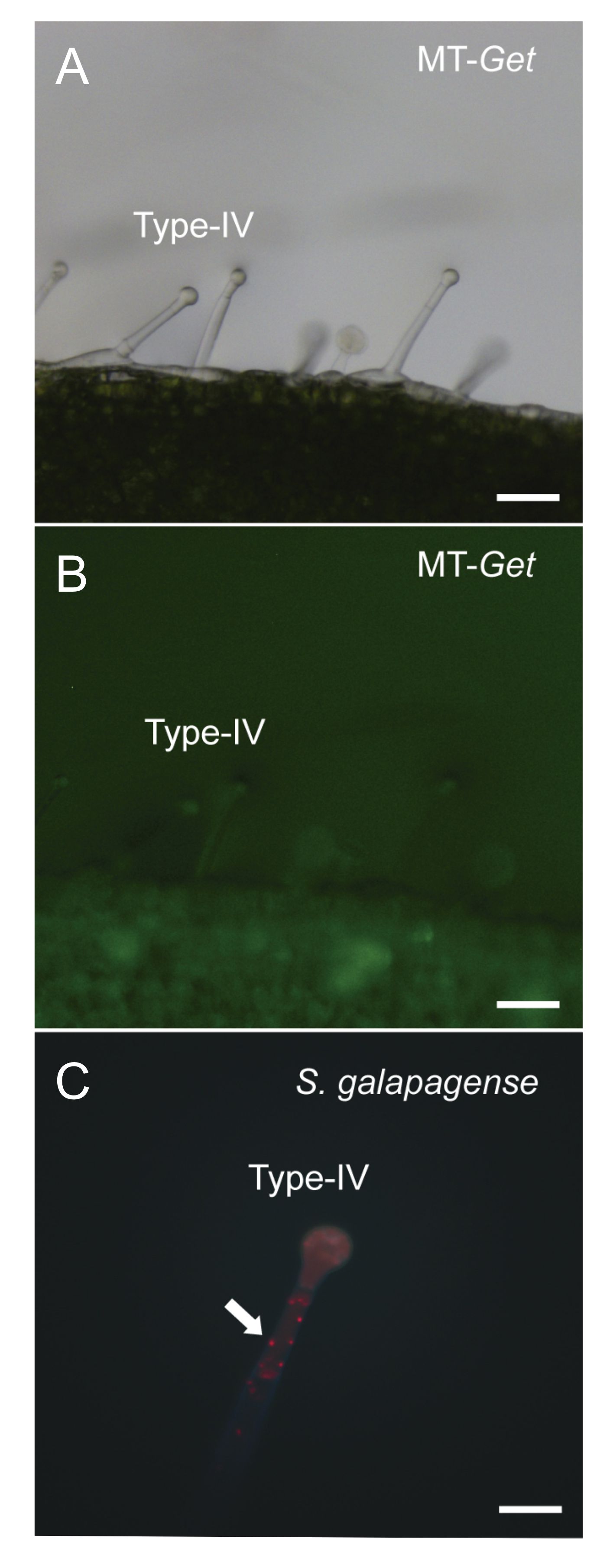

Supplement: Supplementary file 1 [file plants-11-01309-s001.zip › plants-11-01309-s001/Figure S2.jpg]

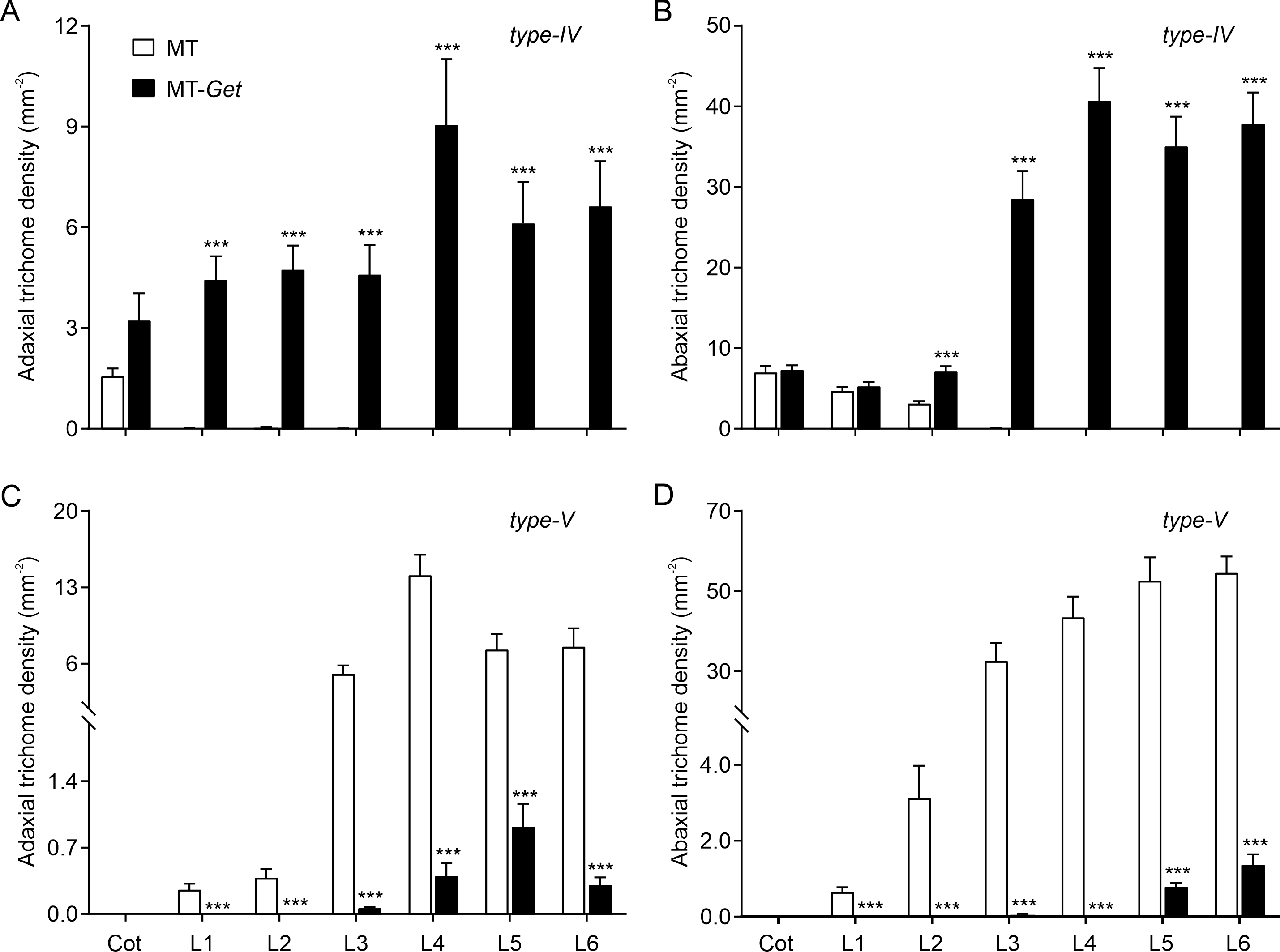

Supplement: Supplementary file 1 [file plants-11-01309-s001.zip › plants-11-01309-s001/Figure S3.jpg]

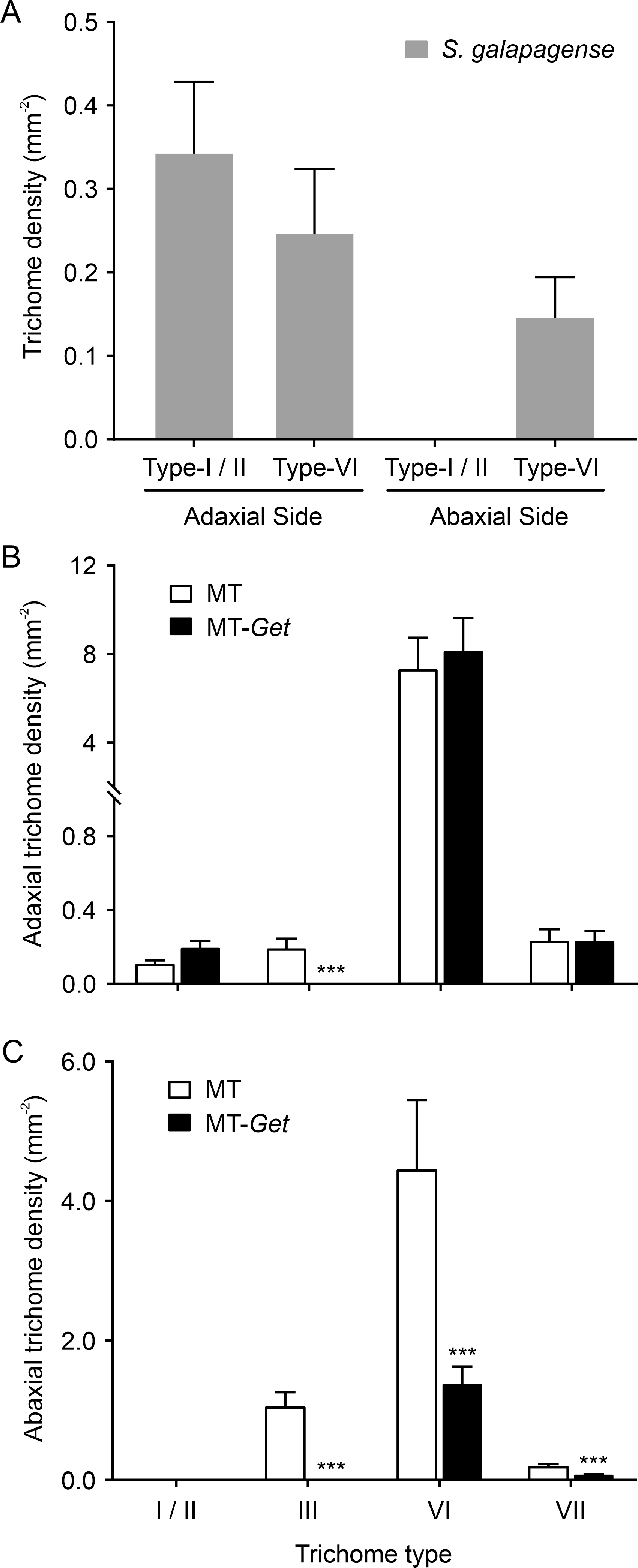

Supplement: Supplementary file 1 [file plants-11-01309-s001.zip › plants-11-01309-s001/Figure S4.jpg]

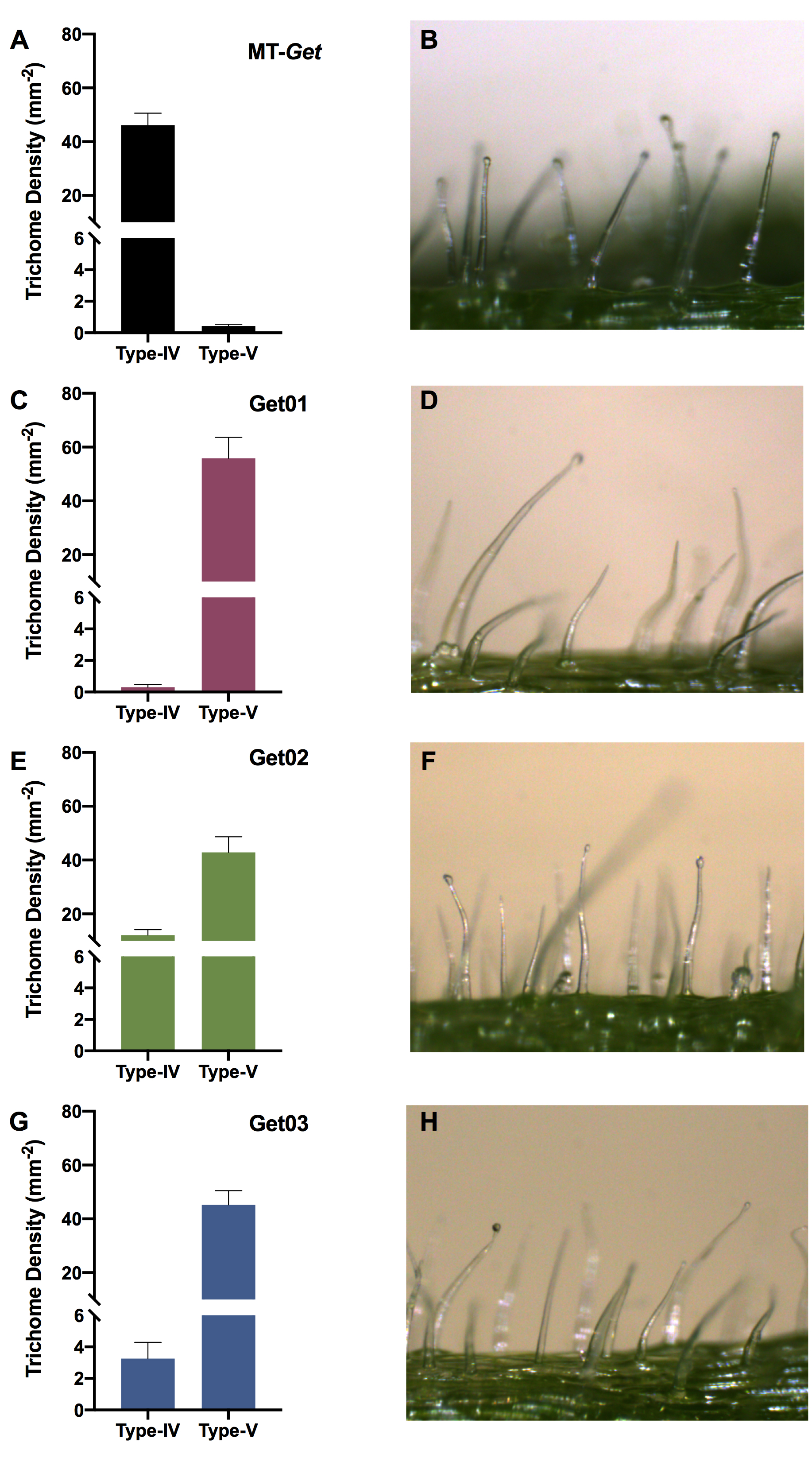

Supplement: Supplementary file 1 [file plants-11-01309-s001.zip › plants-11-01309-s001/Figure S5.jpg]

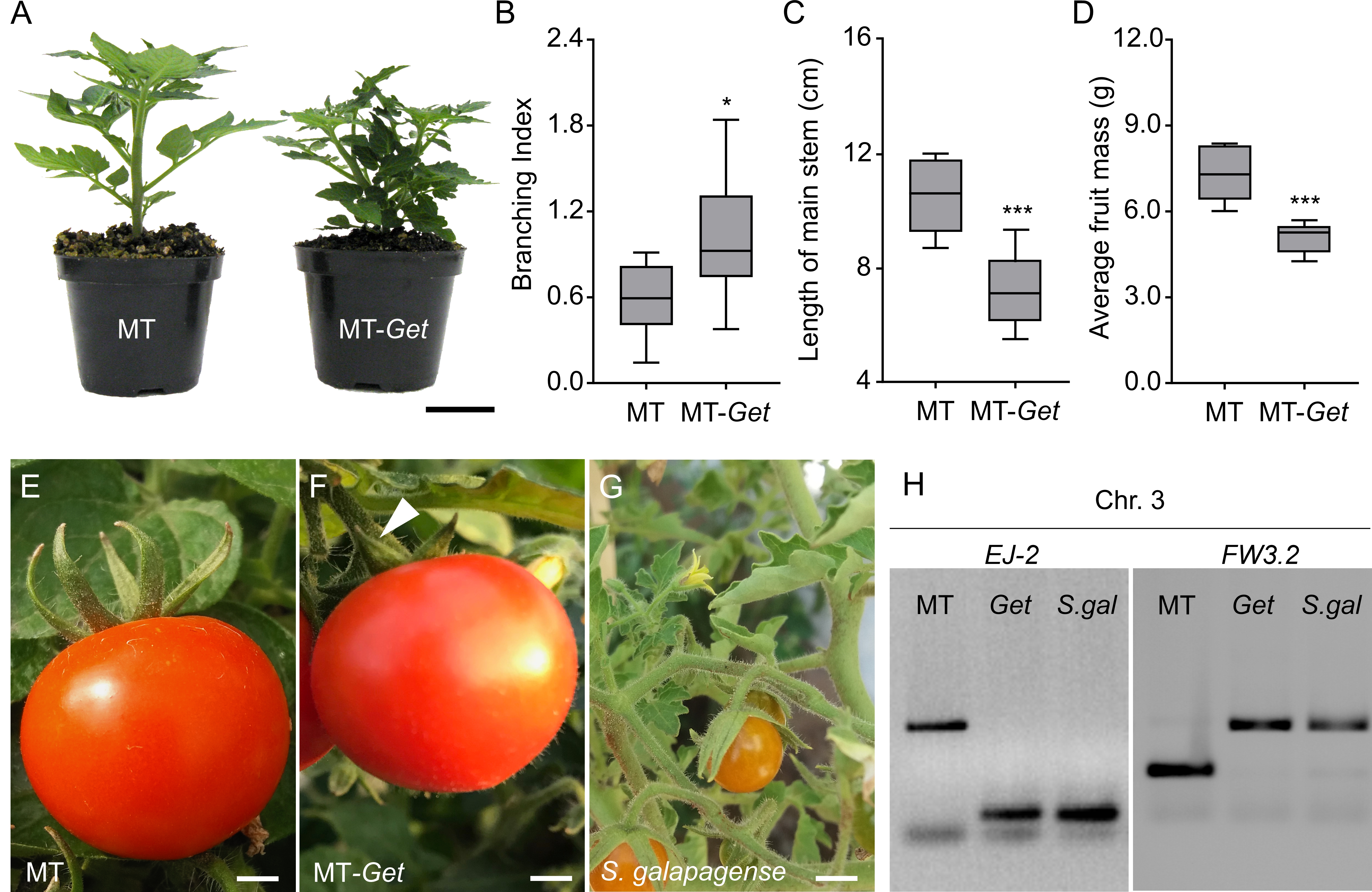

Supplement: Supplementary file 1 [file plants-11-01309-s001.zip › plants-11-01309-s001/Figure S6.jpg]

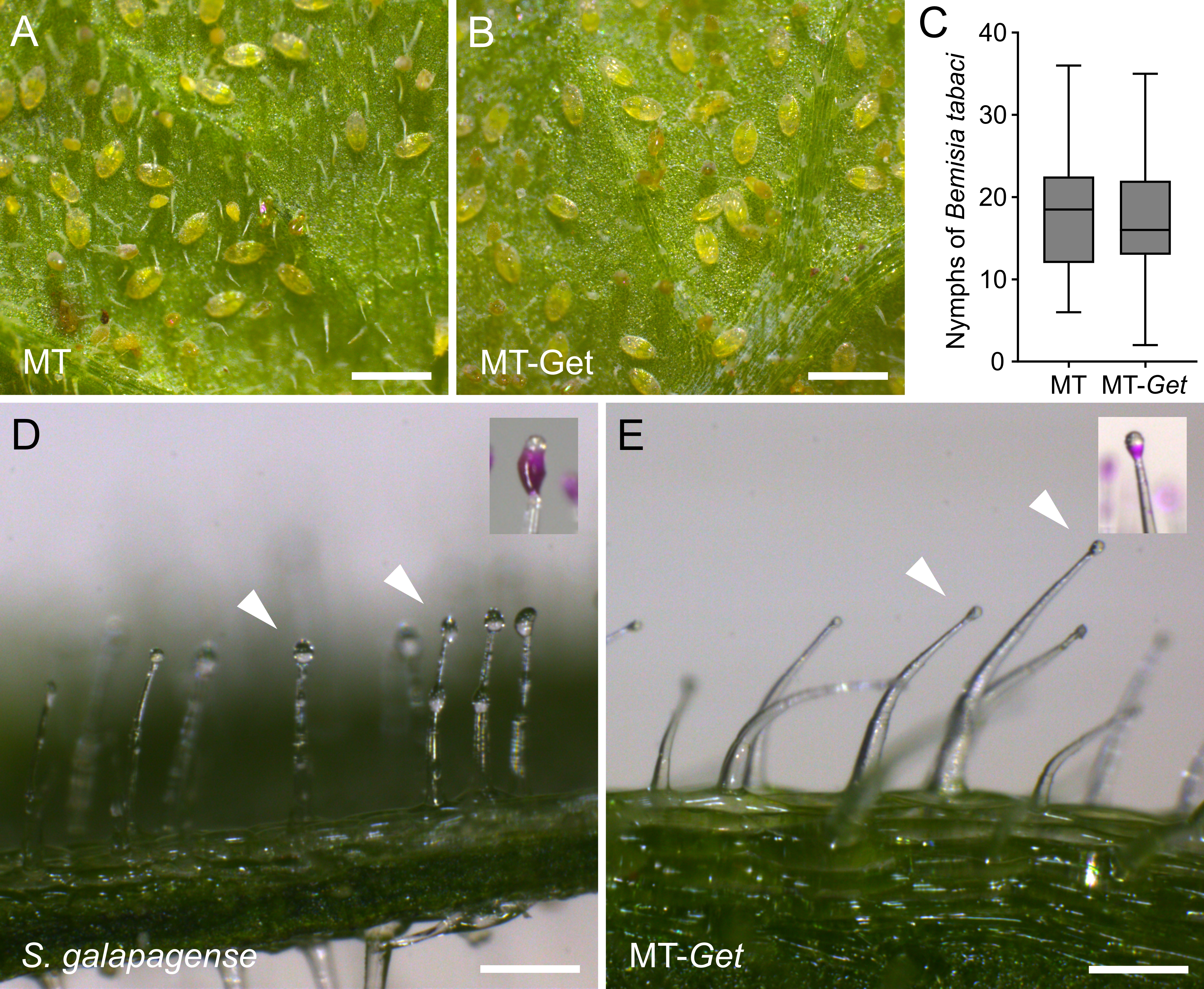

Supplement: Supplementary file 1 [file plants-11-01309-s001.zip › plants-11-01309-s001/Figure S7.jpg]

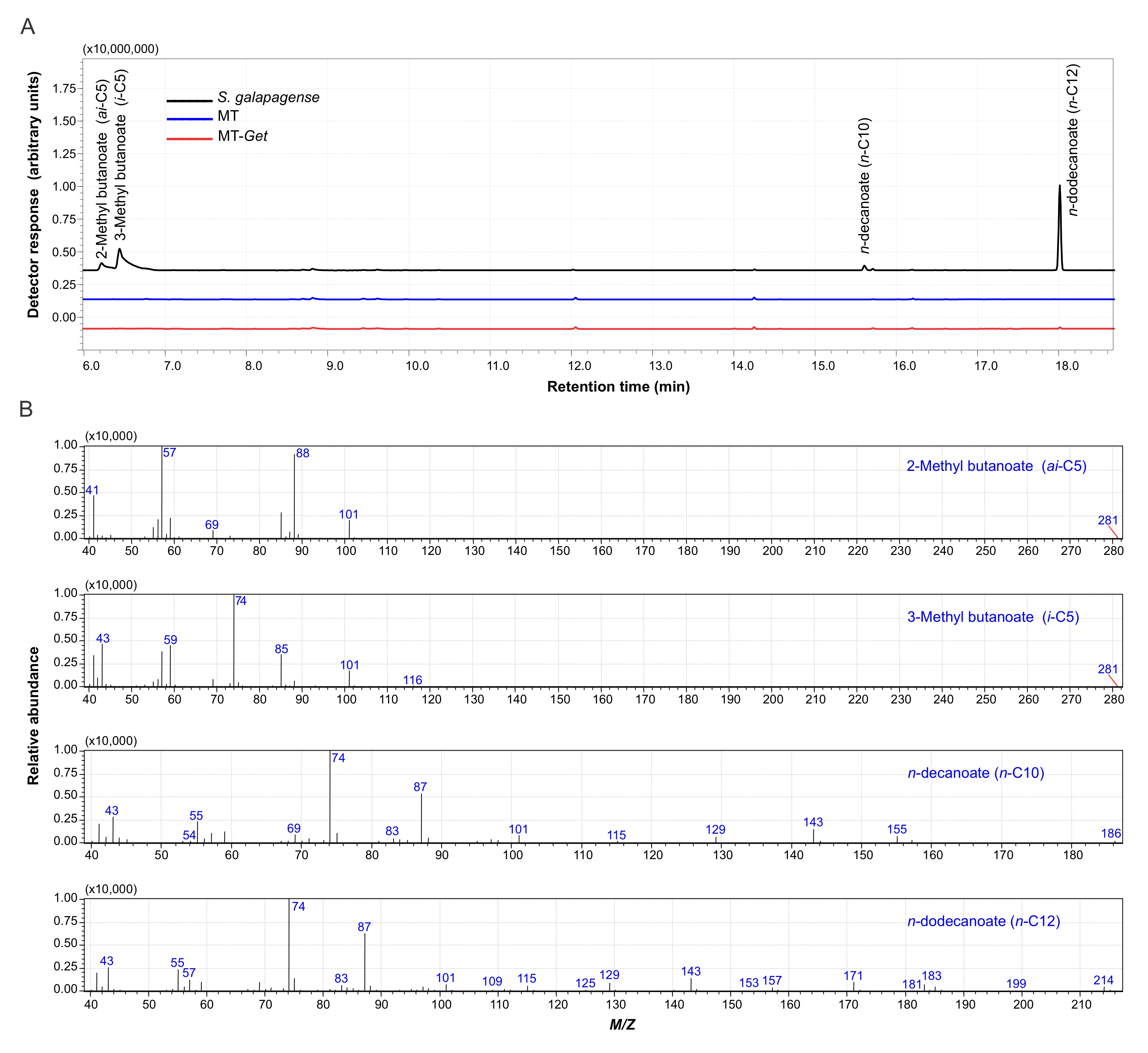

Supplement: Supplementary file 1 [file plants-11-01309-s001.zip › plants-11-01309-s001/Figure S8.jpg]

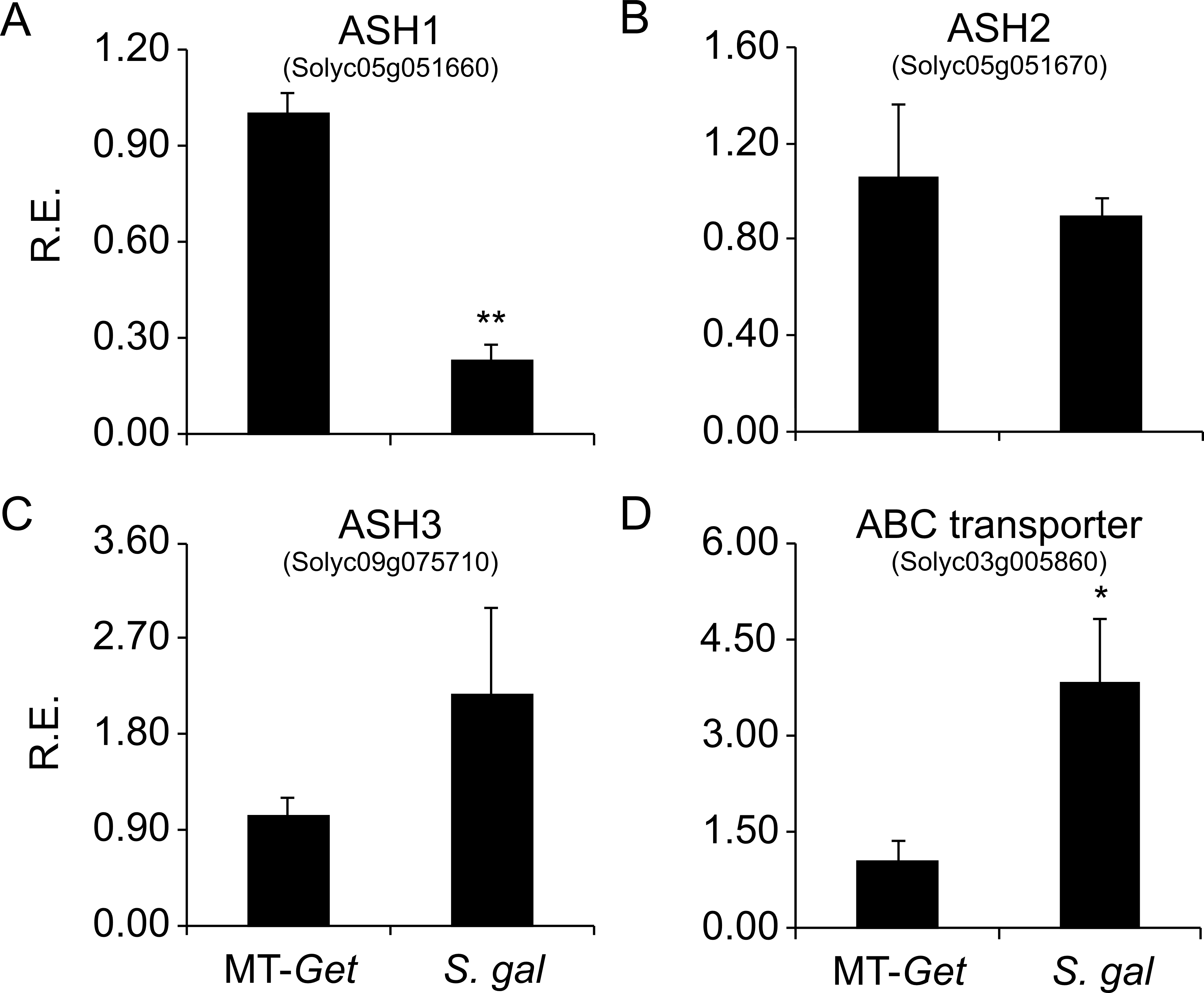

Supplement: Supplementary file 1 [file plants-11-01309-s001.zip › plants-11-01309-s001/Figure S9.jpg]
